# Progress Toward the Implementation of General Health Policies in Iran

**DOI:** 10.34172/aim.31958

**Published:** 2024-12-01

**Authors:** Haniye Sadat Sajadi, Maryam Nazari, Najmeh Bahmanziari, Reza Majdzadeh

**Affiliations:** ^1^Knowledge Utilization Research Center, Tehran University of Medical Sciences, Tehran, Iran; ^2^University Research and Development Center, Tehran University of Medical Sciences, Tehran, Iran; ^3^Department of Health Management, Policy and Economics, School of Public Health, Tehran University of Medical Sciences, Tehran, Iran; ^4^Interdisciplinary Research and Practice Division, School of Health and Social Care, University of Essex, Colchester, UK

**Keywords:** Evaluation, Health policy, Implementation, Iran

## Abstract

**Background:**

Monitoring and evaluation are crucial in ensuring the effective implementation of health priorities. This descriptive study examined the progress towards implementing Iran’s General Health Policies (IGHP) to illustrate how countries can effectively monitor and evaluate their national plans. Additionally, the study sought to identify factors that impede the full implementation of these policies.

**Methods:**

Available data sources, formal reports, and studies were examined to gather data on selected indicators. Then, documentary analysis and 21 semi-structured interviews were conducted to identify measures taken to materialize IGHP and factors that hindered the full implementation of IGHP. Data were analyzed using the content analysis method.

**Results:**

The results showed that several indicators improved during these years, while there was no data for some indicators. There are some barriers to implementing the IGHP, including lack of full understanding of the policies, absence of necessary mechanisms and infrastructures, lack of coherency and alignment of national health plans and policies, absence of monitoring and evaluation framework, and lack of transparency and accountability in the health system. As countries continue to develop their health plans and policies, lack of clarity regarding the progress of these plans remains a concern.

**Conclusion:**

Countries need to strengthen their health planning systems and expedite the implementation of accountability mechanisms within the health system. Enhancing capacity building is essential to establish a comprehensive monitoring and evaluation framework. By fortifying these systems, countries will be better equipped to measure and track progress toward achieving their health objectives.

## Introduction

 A health policy refers to a set of decisions or commitments aimed at achieving a defined health system’s goals. It is assumed to incorporate courses of action (and inaction) that impact the institutions, organizations, and funding arrangements within the health system.^1,2^ Making the right policy is an element of good governance. It leads to defining a vision for the future, which helps establish targets for the short and medium term. Additionally, it outlines priorities and the expected roles of different stakeholders, fostering coalition-building.^3^ These policies and related documents (e.g. strategies and plans) serve several purposes: They respond to calls for strengthening health systems to achieve universal health coverage (UHC), direct the entire pluralist health sector, consider social determinants of health and interactions between the health sector and other societal sectors; and play a key role in enhancing development effectiveness.^4^

 Given the recent focus on national health planning, numerous countries have relied on the formulation of health policies, strategies, and plans for decades to guide and unify their efforts in enhancing health.^5-9^ Iran has made significant efforts to prioritize health issues and needs in various plans. For example, Article 29 of the Constitution of the Islamic Republic of Iran states that providing social security related to retirement, unemployment, old age, disability, accidents, and health needs through insurance is a universal right.^10^ Furthermore, the main directions and priorities of the health sector were included in the National 5-year Development Plans. Iran’s Vision 2025, Iran’s Health Innovation and Science Development Plan, Iran’s Health System Plan, and Strategic planning documents of the Ministry of Health and Medical Education (MOHME) are other examples of national policies and plans that show the future direction of health.

 One of the main national upstream documents in terms of the long-term health targets is Iran’s General Health Policies (IGHP). IGHP is a set of 14 long-term health targets, aligning with international commitment (e.g. Sustainable Development Goals (SDGs) by 2030).^11^ They were notified in 2014 by Iran’s Supreme Leader, who, according to the Islamic Republic of Iran’s constitution, is responsible for outlining the country’s general policies.^12^ The IGHP aim to provide the country with a platform to achieve the health-related goals of Iran’s Vision 2025.

 Monitoring and evaluation (M&E) are crucial to guarantee that health priorities specified in IGHP are effectively executed according to defined objectives and desired outcomes.^4^ They allow us to track the progress of national health strategies, monitor health inequities, and serve as a basis for policy dialogue. Evaluations assess whether the desired outcomes of IGHP have been achieved, helping to identify overall progress, highlight issues, and guide corrective actions. Rigorous M&E generates evidence on the impact of health interventions, aiding better evidence-informed decision-making.^13^

 It has been about nine years since IGHP were launched. Although the country’s main long-term health targets have been defined in this document, no comprehensive assessment of the extent of progress of IGHP has been made. This study aimed to address this gap by assessing the progress in implementing IGHP and identifying the main barriers to their implementation. The findings of this study will be of interest and relevance to different policymakers and authorities who may be interested in finding required actions that may accelerate the achievement of given objectives and improve the health system’s performance.

## Materials and Methods

**Figure 1:**
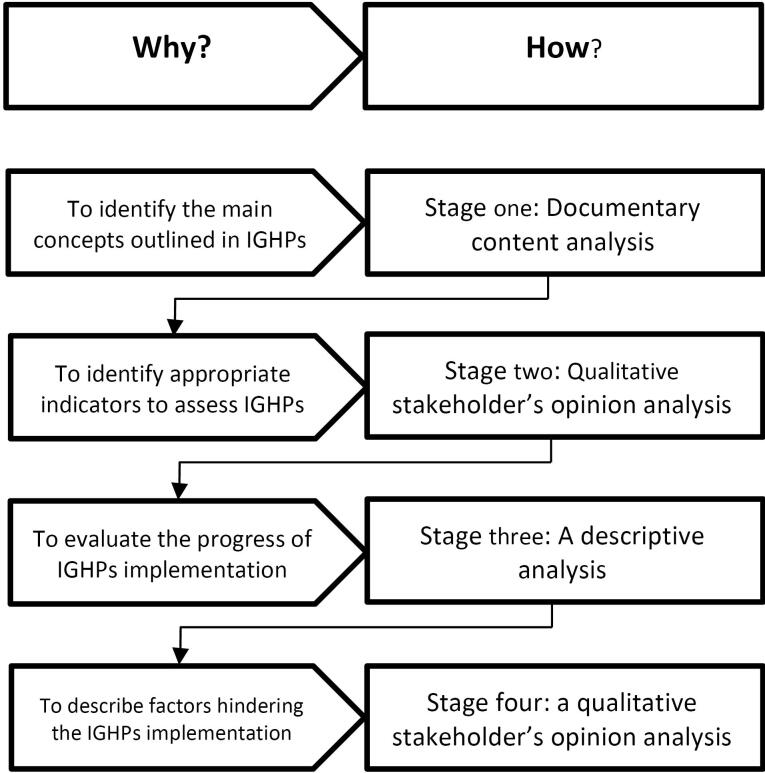
Schematic Diagram of the Study Methodology (Adapted by Authors)

 The current work is part of a study conducted in four stages (Figure 1). The results of the two first stages have been published before. In the first stage, a qualitative documentary analysis was conducted to identify the main concepts outlined in IGHP—containing 14 articles.^11^ Then, another qualitative analysis was undertaken to identify appropriate indicators to assess the IGHP,^14^ and 57 indicators were proposed to assess the progress towards IGHP. In the third stage, a descriptive approach was employed to evaluate the progress of IGHP implementation using the proposed indicators. Given the available data sources and formal reports and studies, data were gathered for at least two points, including 2010 or 2011 or 2012 or 2013 (before), and 2015 or 2016 or 2017 (after starting implementation). Data sources considered for the indicators proposed in this study included health information system, surveys, and censuses.

 At last, a prospective approach using qualitative research methods was employed to describe what has been done to materialize IGHP and identify factors that hindered the full implementation of IGHP. We conducted an extensive documentary analysis on various formal performance reports and research works produced by different individuals and organizations (e.g. MOHME and Health Insurance Organizations). Moreover, we conducted semi-structured face-to-face interviews with key informants to explore what has been done during the last seven years to implement the health targets of IGHP and to find factors affecting IGHP’s implementation. Participants were chosen through purposeful sampling, specifically selected for their expertise and experience in generating and implementing national health policies. Given the homogeneity of the sample and the focused nature of our research objectives, a sample size of 21 was sufficient to achieve thematic saturation, ensuring that no new significant themes emerged during later interviews. The participants’ details are presented in Table 1.

**Table 1:** Participants’ Characteristics

**Participant Characteristic**	**No. of Participants (n=21)**
Gender	
Female	6
Male	15
Age	
Less than 30 years	1
31-40 years	5
41-50 years	9
51-60 years	4
More than 61 years	2
Work experience	
Less than 10 years	1
11-20 years	3
21-30 years	13
More than 31 years	4
Background	
Clinical science	10
Public health	7
Medical education	4
Position	
National policymaker	3
Health policymaker	9
Senior manager	4
Clinician	3
Academician	2

 The interviewees were informed of the results of the IGHP evaluation report. In adherence to ethical principles, interviewers followed a protocol during each interview. This involved introducing the study plan, emphasizing the need for its implementation, obtaining verbal informed consent, and ensuring confidentiality during audio recording. Participants were informed of their right to withdraw at any stage and offered the option to receive final results. To maintain reflexivity, researchers avoided imposing their beliefs or judgments during participant interactions. Additionally, the first author conducted document analysis and interviews. We conducted content analysis to examine the data.

## Results

Table 2 shows the progress toward implementing the IGHP, given the data available for each indicator. Among 67 proposed indicators, there were no data for 20 indicators.

**Table 2:** Trend of Selected Indicators to Measure Progress Toward the Implementation of Iran’s General Health Policies (IGPH)

**Indicator **	**Article of IGHP**	**Relevant Concept in IGPH**	**Before IGHP**				**After IGHP**				**Source **
			**2010**	**2011**	**2012**	**2013**	**2014**	**2015**	**2016**	**Latest**	
Life expectancy at birth (Y)	8, 10, 11	Health status	73	73	74	74	75	75	76	74 (2021)	https://data.worldbank.org/
Probability of dying between the exact ages 30 and 70 years from NCDs^*^ (%)	1, 2	Health status	15.5	15.3	15.3	15.3	15.3	15.4	15.2	14.9 (2018)	https://www.who.int/data/gho
Estimated road traffic death rate (per 100,000)	1, 2	Health status	32.5	27.7	26.1	24.3	22.5	21.8	20.6	21.5 (2019)	https://www.who.int/data/gho
Suicide rate (per 100 000 population)	3	Health status	4.0	3.8	3.5	3.5	3.5	3.4	3.3	3.1 (2019)	https://www.who.int/data/gho
Marriage to divorce ratio	3	Social determinants of health	6.5	6.1	5.5	5.0	4.4	4.2	3.9	2.6 (2019)	https://amar.org.ir/statistical-information
Current expenditure on health per capita (PPP^*^)	5, 10	Health financing	1139	1157	1026	884	998	1003	1147	769 (2020)	https://data.worldbank.org/
Current expenditure on health as a percentage of GDP^*^ (%)	5, 10	Health financing	6.3	6.1	6.1	5.5	6.3	7.2	7.8	5.3 (2020)	https://data.worldbank.org/
Proportion of population using safely managed drinking-water services (%)	6	Risk factors of health	92	93	93	93	93	93	93	94 (2022)	https://www.who.int/data/gho
Prevalence of overweight among adults (%)	6	Risk factors of health	53.6	54.8	56.0	57.2	58.5	59.7	60.9	-	https://www.who.int/data/gho
Prevalence of obesity among adults (%)	6	Risk factors of health	20.3	21.1	22.0	22.8	23.7	24.6	25.5	-	https://www.who.int/data/gho
Population pushed below the poverty line^*^ by household health expenditures (%)	9	Health financing Equity	0.09	0.03	0.04	0.02	0.1	0.08	0.02	0.07 (2021)	https://www.who.int/data/gho
Population with catastrophic health expenditure^*^ (%)	9	Health financing Equity	13.72	14.12	13.87	15.81	15.96	17.03	15.78	15.36 (2021)	https://www.who.int/data/gho
Out-of-pocket payments as % of current health expenditure	10	Health financing	59.40	57.52	54.96	51.07	42.19	44.21	41.40	34.51 (2021)	https://www.who.int/data/gho
Domestic general government health expenditure as % general government expenditure	10	Health financing	12.52	12.04	15.96	16.05	22.60	22.60	21.08	26.11 (2021)	https://www.who.int/data/gho
Public insurance sources of spending on health as % of total health expenditure	10	Health financing	16.0	15.1	17.8	19.7	24.1	28.0	-	-	(15)
Private insurance sources of spending on health as % of total health expenditure	10	Health financing	8.1	8.9	11.9	14.0	11.5	10.6	-	-	(15)
Health insurance coverage (%)	7, 10	Health financing Equity	83.3	-	-	-	-	93.1	-	-	(16)
Iran SCImago rank^*^	1, 14	Introducing innovations in medical education and research	21	17	18	18	16	17	16	15	https://www.scimagojr.com/countryrank.php

NCD: Non-Communicable Diseases, PPP: Purchasing Power Parity, GDP: Gross Domestic Product, Poverty line is the $3.65 a day, Catastrophic expenditure means health expenditure greater than 10% of total household expenditure or income, SJR: Scientific Journal Rankings – SCImago.

 As shown in Table 3, qualitative data indicated that following the IGHP launch, two main reforms were implemented in the country, including the Health Transformation Plan (HTP)^15^ and the Medical Education Evolution and Innovation Plan (MEEI).^16^ Keeping IGHP’s aims and interventions in mind, these two plans were developed.

**Table 3:** Drivers and Barriers of Iran’s General Health Policies (IGHP) Implementation

**Category**	**Sub-category**
Drivers of IGHP implementation	Implementing the Health Transformation Plan Implementing Medical Education Evolution and Innovation Plan
Barriers to IGHP implementation	Lack of full understanding of the concepts, aims, and interventions included in IGHP The absence of necessary mechanisms and infrastructures to fulfill IGHP Lack of coherency and alignment of national health plans, policies, and reforms with IGHP The absence of a monitoring and evaluation framework for IGHP progress Insufficient transparency and accountability across the entire health system

 HTP was proposed to achieve UHC. This plan was a set of instructions to respond to the health system’s critical challenges. HTP received new funding resources, with $3 billion allocated for its implementation in the first year alone. The HTP included multiple interventions aimed at enhancing the sustainability of health financing, expanding health insurance coverage, increasing financial protection, and improving healthcare access and quality.^15^

 “*In simpler terms, when explaining why the health transformation plan was created, they mentioned the general health policies as the legal basis. The MOHME stated that this plan aligned with various important documents, including the general health policies. One of the main aims of the HTP was to decrease out-of-pocket expenses and expand insurance coverage, which is in line with the principles outlined in general health policies. The success of these goals is still uncertain.” *(P. 9)

 In alignment with the responsibilities outlined for the medical education and research system in upstream documents, the primary objectives of this field and the attainable outlook for 2025 have been clarified in MEEI. Subsequently, 12 policies and overarching guidelines were formulated to fulfill the assigned missions, shaping the overall policy direction toward Vision 2025 in higher education and the health system.^16^ A literature review indicated that a comprehensive evaluation of the progress of MEEI has not yet been implemented.

 “*Many clauses in the general health policies focused on medical education and research, with the MOHME prioritizing their implementation. The transformation and innovation packages for medical science education were developed to support these policies and tasks. Additionally, other documents, like the comprehensive scientific map of the country, required implementation.” *(P. 14)

 The qualitative data demonstrated that these two main plans were developed and implemented to materialize IGHP. They had several achievements; however, our findings revealed some barriers to implementing IGHP. These barriers were categorized into five themes.

*Lack of full understanding of the concepts, aims, and interventions included in IGHP:* Participants stated that many efforts have been made to draft and finalize IGHP. Unfortunately, the IGHP were not well-introduced. Most health policymakers and managers were not completely familiar with the content of this document. They forget the main streams of this document over time.

 “*From my perspective, a significant challenge we face in implementing general health policies is the incomplete understanding of the 14 clauses by policy makers and managers. In meetings, I often notice lack of comprehension and awareness of the key points and intentions behind these policies. The original purpose and direction seem to have been lost. Occasionally, they present various cases as part of policy implementation, but due to lack of proper understanding, the efforts become disjointed and sometimes run in parallel.” *(P. 20)

 “*After conducting a survey or test to assess the awareness of general health policies among managers, it is clear that the results are not favorable.” *(P. 3)

*Absence of necessary mechanisms and infrastructures to fulfill IGHP:* Its infrastructure and requirements should be prepared to implement some critical interventions of IGHP. These items were not addressed in the IGHP content. It seems that a feasibility study should be conducted to highlight the prerequisites for IGHP implementation.

 “*Simply having a law in place is not sufficient for implementation. It is essential to have the necessary mechanisms for enforcing that law. For instance, if a law mandates the use of electronic medical records, simply stating it is not adequate. Infrastructure arrangements must be made. While there are commendable general health policies, some lack the required mechanisms and prerequisites for implementation. Therefore, optimism about their execution may be unwarranted.” *(P. 8)

*Lack of coherency and alignment of national health plans, policies, and reforms with IGHPs:* Participants mentioned a gap between those priorities identified by IGHPs and those embedded in national health plans. These resulted in forgetting IGHP directions and moving on to other tracks.

 “*One issue in our country, which also applies to general health policies, is the lack of alignment among our policies, laws, and programs. Essentially, it’s difficult to directly link a goal from the top legal document to the smallest executive plan. While it may sound good in theory, this alignment often falls short in practice. In simpler terms, there is a low level of convergence.” *(P. 12)

*Absence of a monitoring and evaluation framework for IGHPs’ progress:* Most participants cited that the IGHPs did not have a monitoring and evaluation attachment. Moreover, the document did not have a written situation analysis of the health system at the time of announcement. This made it difficult to track the progress of IGHPs.

 “*At the time of notification, general health policies did not have a monitoring and evaluation plan in place. This meant it was unclear which indicators should be used to assess the success of each policy. Subsequently, research was conducted to identify these progress measurement indicators. However, these indicators were not consistently monitored or measured as intended.” *(P. 18)

 “*While we have made strides in health monitoring and evaluation compared to the past, challenges persist in this area. Despite having indicators to track, insufficient data hinders our ability to effectively monitor performance and identify and address issues.” *(P. 6)

*Insufficient transparency and accountability across the entire health system:* Almost all participants highlighted the importance of accountability and transparency in the health system. They recommended that many stakeholders should be involved in implementing IGHPs better and fully. Thus, there is a high conflict of interest among them. There is a need to strengthen accountability and transparency throughout the system to manage this conflict.

 “*Our latest challenge is the lack of a system to hold managers and officials accountable. Currently, there is no mechanism in place to question a manager about their decisions or management practices. While impeachment and retrial exist, they are reserved for very serious cases. We lack a structured way to assess how effectively a manager has implemented policies such as general health policies.” *(P. 8)

 “*For policies to be effectively implemented, transparency is key. It is essential to have a system in place that is open and honest. If we aim for change, we must be transparent about our actions and decisions. By reporting accurately and without censorship, we can identify and address our own shortcomings.” *(P. 19)

## Discussion

 The present study aimed to evaluate the progress of the implementation of the IGHPs and then examine the reasons behind the progress or failure of the implementation of IGHPs.

 Our study identified significant lack of available data for M&E indicators related to IGHPs during the studied period. Out of the comprehensive list of 57 indicators suitable for policy M&E, we could only gather data for a mere 18 indicators. This finding highlights a clear weakness in the recording and collecting of health information. Even the existing health information system in the country, which encompasses routine data and surveys, falls short in calculating data for several crucial indicators. To address these gaps, it is imperative to undertake a comprehensive review and enhancement of the national health information system. This will enable the efficient collection and monitoring of data about key aspects of IGHPs, including tracking progress in promoting equity and professionalism, implementing measures to strengthen governance and service delivery arrangements, and actively involving and empowering the community.^11^ Strengthening the health information system will improve the health system’s overall performance.^17^ Furthermore, it is essential to prioritize enhancing information generation capacity within the health system. It will enable regular and continuous progress monitoring towards achieving UHC.^18^

 Our participants highlighted the lack of a dedicated M&E framework to monitor the advancement of health policies. Without this framework, the targets have not been clearly defined or agreed upon, making it challenging to determine whether the desired progress is being achieved.^4^ Nevertheless, regarding the trend of proposed indicators, our findings indicate limited progress in implementing IGHPs based on the available data. Although certain indicators, mainly related to health status, have shown improvements, these advancements may largely be attributed to the Primary Health Care reform initiatives implemented since the late 1970s.^19^ However, it is crucial to acknowledge that sustaining these improvements presents significant challenges in the face of global threats, such as emerging and re-emerging diseases and the impact of climate change and the country’s specific economic sanctions. Therefore, the health system must possess multiple capabilities to effectively operate across a wide geographical area, especially during critical situations and large-scale crises.^20^

 In addition to the mentioned improvements, areas still require further enhancement, particularly regarding health risk factors and the control of non-communicable diseases (NCDs). Despite notable achievements in preventing and controlling NCDs in Iran, these conditions continue to present significant challenges to the country’s health system.^21^ One of the significant challenges to Iranians’ health is the very high rate of premature death (death under 70 years), which is currently around 50%, mainly due to cardiovascular conditions. Recent studies indicate that cardiovascular disease (CVD) is the primary contributor to premature mortality in Iran, particularly among adults aged 30-69. It is recommended that systematic screening for adults aged over 35 should be implemented to identify cardiovascular risk factors early and prevent premature cardiovascular mortality. Recent trials, such as the PolyIran study in Iran, support this strategy.^22^ Scholars have emphasized the necessity for innovative measures and strategies to strengthen Iran’s health system in NCD prevention. These measures include empowering human resources, promoting effective collaboration within and between sectors, involving non-governmental organizations and charities, and utilizing evidence-based studies in policy and decision-making processes.^23^ It is worth noting that these improvements can be accomplished without relying solely on financial resources.

 The findings also raise concerns about the increasing trend of divorce in Iran, which has become a significant social issue. Previous studies have reported a rise in the divorce rate and have examined the underlying causes. Additionally, the country has implemented programs to control and reduce the divorce rate.^24-26^ Nevertheless, these programs have faced significant challenges, highlighting the country’s need for comprehensive social programs and more investment in health in all policies. The successful implementation of Health in All Policies in Iran hinges on enhancing intersectoral collaboration among key government departments, including health, education, environment, and urban planning. While the MOHME plays a central role in health governance, fostering collaboration among these sectors can facilitate the integration of health considerations into decision-making processes. Regular monitoring to evaluate the health implications of policies in diverse sectors are essential to prioritize health in policy formulation.^27^

 Our findings also revealed the instability of certain measures implemented to reform the financing arrangement and enhance the health system’s resilience, considering that one of the main constraints during these years was economic due to sanctions. The recent fundamental reform in Iran’s health system aimed to improve the sustainability of health financing, expand health insurance coverage, and increase financial protection. However, the instability in financial resources has posed challenges and weaknesses in implementing these initiatives.^28^ Therefore, the efforts to establish a resilient system have been limited. The issue of fair financial contribution has consistently posed challenges in Iran. To tackle this, recommended policies and programs include integrating insurance funds, enhancing sustainable financial resources, targeted allocation of subsidies, prioritizing services, and implementing performance-based payment systems.^29^

 Furthermore, the indicator of total expenditure on health suggests that Iran allocates the same funds as other similar countries towards health care. However, it is important to emphasize the need for sustained public spending and enhancing health system efficiency. The health system’s resilience is heavily influenced by contextual factors such as the economic, social, and political situation, as well as global circumstances like pandemics or armed conflicts. It is especially significant for countries like Iran, which have faced numerous international sanctions and economic shocks.^30^

 The findings also indicated that Iran has advanced medical education and research. However, limited evidence is available to assess the extent of progress in integrating responsive medical education and research into health services. Scholars have reported a lack of sufficient connection between medical education and health service delivery, resulting in challenges regarding the availability, accessibility, acceptability, and quality of the health workforce.^15,31^

 In summary, several barriers hinder the full implementation of IGHPs. These barriers include lack of comprehensive understanding of the concepts, objectives, and interventions associated with IGHPs, inadequate mechanisms and infrastructure to support their implementation, lack of coherence and alignment between national health plans, policies, and reforms, absence of a monitoring and evaluation framework, and lack of transparency and accountability within the healthcare system. Limited evidence suggests additional barriers, such as absence of executive strategies, a national action plan, and information infrastructure, poor management practices, insufficient budget allocation, and failure to address certain national priorities, as identified by other researchers.^32^ To overcome these obstacles and achieve the goals of IGHPs, the nation must demonstrate determination, remove barriers, and renew its commitment to realizing these policies.

## Conclusion

 The progress in implementing IGHPs fell short of expectations. The challenges in partially implementing IGHPs stem from various obstacles. To overcome these challenges, there is a clear need to enhance the health planning system to align goals and programs across different planning levels in response to the priority challenges faced by the health system. It is vital to establish a robust monitoring and evaluation system within the health system to provide policymakers with timely and accurate data necessary for evaluating the effectiveness of each program and policy. Ultimately, expediting the implementation of accountability systems within the health system is essential. It will allow society and regulatory bodies to assess managers’ efforts in executing planned priorities and utilizing allocated national resources transparently.
